# Gossip and coping with social isolation: the case of migrant truck drivers in Western Europe

**DOI:** 10.3389/fpsyg.2024.1334780

**Published:** 2024-06-26

**Authors:** Elena Martinescu, Bianca Beersma

**Affiliations:** Department of Organization Sciences, Vrije Universiteit Amsterdam, Amsterdam, Netherlands

**Keywords:** gossip, social isolation, coping, job-demands resource model, truck drivers

## Abstract

This article examines how employees use gossip as a resource to cope with social isolation. Building on a qualitative study with 32 truck drivers in a Western European company, our research identified gossip in close relationships and gossip in distant relationships as distinct patterns playing a different role in coping with social isolation, and a third pattern in which gossip was not beneficial. First, gossiping with close friends at work helped drivers engage in emotion-focused coping by reducing stress and loneliness. Second, gossiping with distant colleagues helped drivers engage in problem-focused coping by exchanging knowledge involving people in the organization. Third, gossip avoidance occurred in distant relationships, where drivers limited gossip exchanges going beyond instrumentally useful information. Overall, these findings show that drivers relied on different layers of their social network to cope with social isolation. Enriching previous research, this study shows that gossip represents an essential resource for emotion-focused and problem-focused coping.

## 1 Introduction

Recently, the work of truck drivers has captured public interest and has been featured in mainstream news such as CNBC or The Guardian (Cerullo, 2022; Kale, 2022). An article in The New York Times described life as a truck driver to be a dystopian nightmare: “*Just imagine finishing 10 h at a desk job, only to return to your apartment to find the heat did not work. That’d be quite frustrating. Then imagine your apartment was your office and most nights dinner was a microwaveable burrito or a bag of fast food. And then imagine your desk job required you regularly press a little pedal, you could not stand up, you had essentially no face-to-face contact with co-workers, and if a bathroom did not easily present itself you were forced to use a plastic jug — all while a computer or a person at a desk hundreds of miles away monitors your every move*” (Kaiser-Schatzlein, 2022).

As illustrated above, long-distance truck drivers face particular challenges in their work. Because the job design maximizes technical efficiency of equipment and input of employees, divers may be on the road for several weeks at a time, without the possibility to return to their family home or to a company location. Drivers spend most of their time alone, with few opportunities to meet coworkers or supervisors for lengthy periods of time. Therefore, due to the nature of the job, one of the most taxing aspects of truck drivers’ work is social isolation (Apostolopoulos et al., 2016). Social isolation represents a lack of contact with other people and being disengaged from groups and social activities (Taylor, 2020), and in the workplace it is associated with being physically distant from others (Bartel et al., 2012). Social isolation in the workplace is problematic because it has detrimental effects for well-being, job satisfaction, and organizational commitment (Kurland and Cooper, 2002; Marshall et al., 2007). Research has shown that among truck drivers, loneliness and strain on family life is a primary cause of stress and employee turnover (Williams et al., 2017).

Although long distance truck drivers face social isolation at work (Apostolopoulos et al., 2016) it is currently not well understood how drivers cope with this aspect of their job. Due to working in isolation truck drivers are likely to experience a high need for social contact with others in order to reduce feelings of loneliness (Baumeister and Leary, 1995; Apostolopoulos et al., 2016). Anecdotal evidence from a recent interview with a truck driver in The Guardian shows that indeed truck drivers do keep in touch with other drivers regularly: “*Lorry drivers tend to speak about one of five things: routes, traffic, schedules, other drivers and food. If Piper discovers a useful tidbit – perhaps […] a client told him something interesting as they were unloading his lorry – he passes it on to Alan and Lloyd carefully for inspection, like a piece of ancient pottery at a dig. ‘There is a lorry-driver grapevine,’ Piper says. This camaraderie – the inane, meandering chats – makes the job tolerable. Lorry driving is lonely work and the job has an emotional price”* (Kale, 2022).

Thus, truck drivers may spend a significant amount of time each day talking with their colleagues in order to mitigate the effect of social isolation; moreover, as suggested above, drivers often share gossip through the “lorry-driver grapevine.” This is not surprising, because gossip has been shown to be omnipresent in conversations across different social contexts (Emler, 1994; Dunbar et al., 1997; Dunbar, 2004; Foster, 2004). Scholars report that people spend over 65% of their time in day-to-day conversations gossiping (Emler, 1994; Dunbar et al., 1997; see also Goldsmith and Baxter, 1996). Gossip seems to be a universal human behavior, intrinsic to social and organizational life (Dunbar, 2004).

Relying on findings of a recent systematic review of the gossip literature, we adopt a broad definition of gossip, according to which gossip represents communication between a sender and a receiver about a third person who is absent, i.e., gossip target (Foster, 2004; Dores Cruz et al., 2021). This scientific definition of gossip includes all conversations in which people discuss about someone who is absent, and allows for variation on gossip valence (i.e., from negative to positive), level of formality, confidentiality, topic, and motive for sharing gossip. Accordingly, gossip may be about a person’s characteristics, behavior, roles, social relationships, or events they are involved in, as recently shown in a study where participants reported gossip they had heard in a work context (Martinescu et al., 2022).

Although gossip has not been studied as a resource for coping with challenges derived from workplace social isolation, previous research shows that gossip functions as a resource to fulfill emotional or informational needs (e.g., Martinescu et al., 2019). Gossip plays an important role in emotion regulation and social bonding; more broadly, gossip fosters information exchange, and learning vicariously about one’s social environment (e.g., Fine and Rosnow, 1978; Baumeister et al., 2004; Foster, 2004; Bosson et al., 2006; Beersma and Van Kleef, 2012; Wu et al., 2016). As such, engaging in gossip could be beneficial for coping with social isolation.

In this paper we explore the role gossip may have in coping with challenges arising from workplace social isolation. Drawing on the Job-Demands-Resources Model (Demerouti et al., 2004; Schaufeli and Bakker, 2004; Bakker and Demerouti, 2017) we conceptualize social isolation as a job demand, and gossip as a resource employees may use to cope with this demand. Specifically, we examine the role of gossip in coping with challenges that drivers face due to their socially isolating work using a qualitatively grounded approach (Strauss and Corbin, 1998). In particular, we aim to understand drivers’ social reality and lived experiences of social isolation, and their own representations of how these are connected with gossip. In doing so, we offer two key contributions to the existing literature. First, this study contributes to a better understanding of coping strategies in a socially isolating work context, which existing studies do not accurately describe, predict, or explain. We extend theorizing about the use of gossip as a coping mechanism with social isolation in the workplace. Second, a qualitative approach in this study adds a novel perspective on gossip research, by revealing the meaning drivers give to their experiences of gossip.

The work of long-distance truck drivers is worthy of attention for a number of reasons. First, as an extreme example (Eisenhardt, 1989), it allows a clear identification of challenges arising from social isolation, and exploration of ways in which gossip helps cope with these than in other types of work where isolation is less acute. Second, long-distance truck drivers have an essential role in the supply chains of a functioning economy (Wilson, 2015). However, in recent years there have been notable shortages of qualified truck drivers around the world, and this problem is estimated to worsen in the future (IRU, 2022). Against the backdrop of a global shortage of truck drivers, it is important to understand how they cope with job demands in order to improve well-being and retain qualified drivers in their jobs for longer. Third, as an occupational group, truck drivers face particularly high risks of comorbidities, such as cardiometabolic disease (Hege et al., 2017), accidents (Apostolopoulos et al., 2016), and maladaptive behaviors such as drinking and use of stimulant drugs (Raggatt, 1991). As such, this study reveals what kind of support drivers might need in order to improve their safety, health, and wellbeing.

## 2 Theoretical background

According to the Job-Demands-Resources Model (Demerouti et al., 2004; Schaufeli and Bakker, 2004; Bakker and Demerouti, 2017), job demands are requirements a job places on the individual, with associated physiological and psychological costs, whereas job resources are physical, social, psychological, material or organizational aspects of a job that enable individuals to fulfill work goals, reduce job demands, or stimulate growth or well-being. The JD-R model proposes that job demands and resources have unique and independent effects on well-being. Prolonged exposure to job demands initiates a health impairment process, through chronic exhaustion and burnout, whereas job resources initiate a motivational process, by providing meaning and satisfying basic needs, and facilitating work engagement (Bakker and Demerouti, 2017). Importantly, job resources can buffer the effect of job demands on strain. As such, job resources enable employees to cope with job demands. Furthermore, a wide range of job resources can counter the effect of demands on wellbeing. The JD-R model has a broad scope and is heuristic in nature, as it facilitates thinking about how job characteristics (i.e., demands and resources) affect employee wellbeing (Schaufeli and Taris, 2014). Because the JD-R model is non-limitative in terms of the study concepts, and can be tailored to the specific needs and situation experienced by employees of an organization, we deem the JD-R appropriate as a theoretical framework for examining how drivers may use gossip as a resource to cope with social isolation.

Social isolation is a job demand that depletes long distance truck drivers’ emotion regulation resources (Demerouti et al., 2004; Schaufeli and Bakker, 2004; Apostolopoulos et al., 2016) and requires engaging in coping behaviors to reduce its negative effects (Raggatt, 1991; Hege et al., 2017; Williams et al., 2017). Coping represents the cognitive and behavioral efforts made in order to manage demands that exceed one’s resources (Lazarus and Folkman, 1984). Because gossip provides emotional support and access to information (e.g., Fine and Rosnow, 1978; Beersma and Van Kleef, 2012; Martinescu et al., 2019), gossip may help drivers cope better with the effects of social isolation.

First, gossip facilitates social bonding and social support between the people who engage in it. By exchanging gossip with colleagues, employees are likely to discover that they have similar perceptions or attitudes toward the gossip target, and this leads to a sense of shared reality and increases trust in one’s gossip partner (e.g., Fine and Rosnow, 1978; Bosson et al., 2006; Peters et al., 2017). Furthermore, through gossip, people can share their experience of interacting with others, and expressing their concerns. A gossip conversation can offer a safe way of venting negative emotions and getting social support from one’s conversation partner (Foster, 2004; Waddington and Fletcher, 2005; Bosson et al., 2006; Peters and Kashima, 2007; Peters et al., 2009; Rimé, 2009). As such, by providing social bonding and emotional support, engaging in gossip could help reduce emotional costs of social isolation.

Second, engaging in gossip helps members of (organizational) groups to access and validate information about others. Information obtained through gossip can help people get to know others in their network, make sense of their social environment, and understand its norms (Fine and Rosnow, 1978; Baumeister et al., 2004; Beersma and Van Kleef, 2012; Martinescu et al., 2019). By collecting information through gossip, people can compare their observations and understand the people around them (Wert and Salovey, 2004). Gossip can help people decode the behaviors of others and more accurately understand their intentions and trustworthiness (Mills, 2010; Beersma and Van Kleef, 2012). Moreover, exchanging gossip helps individuals learn about the consequences of other’s behavior (e.g., breaking norms), and this knowledge can provide useful insights they can apply to their own lives (Martinescu et al., 2014). Thus, by facilitating access to information about others, gossip could help drivers fill information gaps they face due to working in social isolation. As shown by previous research, employees engage in informal knowledge sharing when they lack the organizational structures and procedures for doing so (Taminiau et al., 2009), which might be the case for people working in social isolation.

To summarize, gossip may be a useful resource in coping with consequences of social isolation. Therefore, we conducted this study with the goal of exploring in a qualitative manner how employees in a socially isolating job may use gossip to cope with this job demand.

## 3 Methods

To develop an understanding of the role of gossip in coping with social isolation, we used an interpretivist approach, which involves an iterative process of moving back and forth between data Research Topic, analysis, and theorizing (Strauss and Corbin, 1998). Qualitative research methods are well suited to developing new theory about complex processes such as coping with social isolation. In particular, qualitative methods highlight the lived experiences of individuals and draw meaningful insights from these. By identifying patterns in lived experiences of individuals, and understanding the meaning people give to their experience in their current context, qualitative research does not have high generalizability, but is valuable in generating novel insights that provide a basis for new hypotheses to be tested with quantitative methods.

### 3.1 Research context

We conducted a qualitative study in a distribution company employing long distance truck drivers. We conducted this research at GTR (a pseudonym), a company based in Western Europe, which employs most of its drivers from Eastern European countries (over 300 drivers). Drivers are usually assigned several deliveries per day (typically 2–4), with destinations within Western Europe. Drivers work in shifts of 3, 6, or 9 weeks, and have a following break of 1, 2, or 3 weeks, respectively, which they usually spend in their home country. Drivers work from Monday to Friday and have a high workload, with workdays taking up to 15 h, of which a maximum of 10 h represent effective driving time, in accordance with the EU legislation. During their shifts, drivers live in the company truck on the road, and can occasionally spend their weekend break at one of the few company bases. Work tasks are assigned by a delivery planner through an on-board IT system designed to automate communication between drivers and the delivery planners and minimize contact with the management. Lastly, the job entails no interactions with other drivers, and few foreseeable opportunities to meet colleagues in person. Therefore, drivers are socially isolated for an extended time from their colleagues as well as from families and others in their home country.

### 3.2 Data collection

After receiving approval for the study from the university’s Ethics Review Board, we conducted semi structured interviews with 32 male drivers from 6 Eastern European countries (3 EU, and 3 non-EU). Mean age was 42.9 years (*SD* = 8.06), and average tenure was 4.32 years (*SD* = 3.61). An overview of respondent demographics is presented in the Table 1. Interviews were conducted with drivers during their weekend break; 26 interviews were conducted face-to-face on a company base, and 6 through video-calls. Interviews were conducted by the first author in English or in another language the interviewer and participants spoke fluently^1^. Typically, interviews lasted 30–60 min, were audio recorded and later transcribed verbatim and, if not already in English they were translated into English. Drivers participated voluntarily, were informed that their data would be anonymized after the interview and gave their written consent for participating in the study. Although we did not pre-select drivers on any criteria except speaking a common language with the interviewer, the participant sample was diverse regarding nationality, age, and tenure in the company, allowing us to identify and define emergent theoretical categories and patterns.

**Table 1:** Participant demographics.

Respondent	Nationality	Age	Tenure (years)
1	C&D	50–54	13
2	B	45–49	3
3	B	45–49	2.5
4	B	50–54	3
5	C&D	45–49	10
6	E	40–44	3
7	E	35–39	1
8	E	30–34	3
9	D	30–34	3
10	B	45–49	1
11	E	35–39	3
12	E	35–39	1.5
13	D	35–39	3
14	D	50–54	1
15	C&D	45–49	4.5
16	C	45–49	4
17	F	50–54	12.5
18	D	45–49	9.5
19	C&D	40–44	12
20	D	40–44	4
21	C&D	40–44	7
22	D	40–44	6
23	C&D	40–44	4
24	D	55–59	0
25	C	45–49	3
26	E	25–29	2
27	D	60–64	9
28	A	30–34	2.5
29	D	35–39	2.5
30	D	35–39	2
31	E	30–34	2
32	E	35–39	1

The interview protocol was guided by the question of “What do respondents gossip about with their colleagues, and for what purpose?.” Although our *a priori* research question was aimed at understanding the role of gossip in coping with social isolation among drivers, the semi-structured interviews allowed the emergence of other insights related to the use of gossip in this context (Gioia et al., 2012).

At the start of the interview, drivers were asked to describe their role in the company and a typical day at work, and then to describe their perception of the company and its culture. These questions were intended to set the ground for the gossip related questions, which followed afterwards, or to probe for gossip. According to the semi-structured interview protocol, participants were asked about their gossip exchanges and the context of gossip conversations. Specifically, they were asked with whom they talked most often at work and why, what were typical subjects of conversation, if and how they received news about other colleagues (i.e., gossip targets), if they talked about their colleagues, superiors, and clients with others (i.e., gossip), and how these gossip conversations influenced them (if at all). Drivers were also asked if there was anyone they avoided talking to, and why. When drivers reported that they did engage in gossip, the interviewer addressed follow-up questions. These questions asked respondents to think of a concrete example of sharing or receiving gossip, and reflect on their own or others’ motives, the context, and on how the gossip influenced their behavior or made them feel. Data Research Topic was considered complete when interviews did not provide any new information (i.e., data saturation); the responses provided sufficient and recurring input to form a solid basis for our findings.

### 3.3 Analysis

The interviews were analyzed using a thematic analysis approach, using the Gioia method (Gioia et al., 2012). In line with these recommendations, we started with the data analysis after the first author conducted the first few interviews. Furthermore, the authors met regularly to discuss coding decisions, emerging findings, how the interview questions should change with the progression of the research, and the theoretical approach. We used the software ATLAS.ti, a tool facilitating qualitative data coding.

In a first step, interviews were read several times, and open codes were created to descriptively capture accounts of gossip between drivers (e.g., keeping in touch with friends, talk about planners) and the motive for engaging in gossip (e.g., to seek emotional support, to exchange information, to vent). Accounts resulted from participants’ descriptions of why and how they had engaged in gossip, or what gossip they had heard from others. The authors discussed these episodes of gossip and their descriptive codes.

Second, we further explored emergent findings and grouped the first order codes into broader explanatory categories by seeking similarities and differences among the codes (i.e., axial coding). During the coding process we re-read interviews to check whether our understanding of the categories that emerged matched the ideas expressed by participants, and re-coded or dropped categories if necessary. The categories created in this step reflected different functions of gossip (e.g., maintain bonds, protect others), based on literature describing gossip functions and motives (Dunbar et al., 1997; Foster, 2004; Beersma and Van Kleef, 2012), and types of relationships between gossipers (Ellwardt et al., 2012).

**Figure 1:**
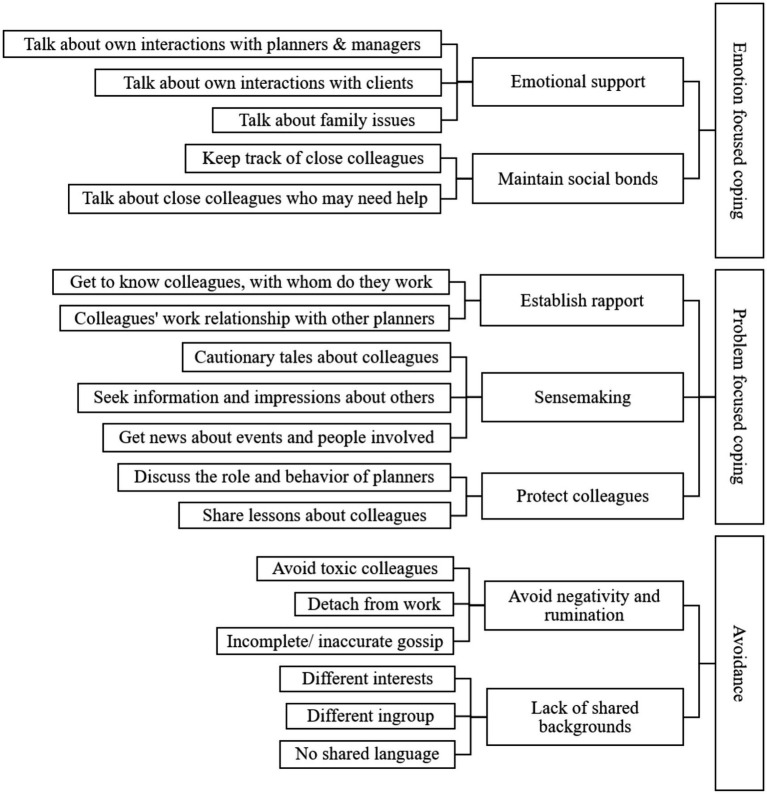
Coding tree for gossip behavior among truck drivers.

In the third step of coding we used previous categories to identify aggregate dimensions that demonstrate how gossip was used as a resource for coping with social isolation. We focused on theorizing about the role of gossip as a coping strategy, based on Lazarus (1991), who distinguishes two patterns of coping. Emotion-focused coping is aimed at regulating one’s negative emotions caused by perceived harm or threat, for example by sharing one’s stressful experience with others and seeking social support. Problem-focused coping is aimed at changing one’s relation to the environment, such as seeking information that can be used to eliminate the source of stress. Using this distinction, we conducted a conceptual categorization and integration of previous codes in order to identify gossip patterns among drivers and their use as coping strategies. The data structure is presented in Figure 1.

## 4 Findings

### 4.1 Social isolation

From the first interviews it emerged that respondents indeed experienced social isolation, as we had anticipated based on previous research (Apostolopoulos et al., 2016; Williams et al., 2017). This is illustrated below.

*To stay 9 weeks, to stay so long isolated, that is too much. I'm telling you I wouldn't do this again. […] Because it happens that you can't handle it anymore after some time. And then something [bad] can easily happen. It’s very stressful. […] I know a lot of people who just collapsed, they couldn't cope, mentally. You accumulate too much pressure (R9).*

Drivers also reported that they had difficulties being away from loved ones and feeling disconnected from their friends and communities at home:

*I'm separate from my family. That's the biggest problem for me (R7).*

*Only you on the truck, you lose your friendships. You start growing dumber. […] you start growing like an animal because you don't have a connection with the people; you only have a connection with the animals. And when you don't share the bad ideas and the bath thinking, what pain they have, what problem they have, this is growing in your body, it's in your brain (R2).*

*[You are not] the person who goes home on Friday, and has interactions with others, and has a house [… you make] this sacrifice, to live semi-homeless, 2 months in a cabin (R20).*

In summary, drivers experienced high social isolation at work. In the sections below we present findings regarding the role of gossip in coping with social isolation.

### 4.2 Gossip with close friends: emotion focused coping

Drivers reported that they had a small circle of close friends at work, with whom they have a shared background (e.g., people from the same country or region, colleagues from previous companies). Within these close relationships, gossip conversations were focused on emotional connection with others, fostering a sense of community, as well as relaxation and enjoyment.

#### 4.2.1 Emotional support

Drivers reported feeling socially isolated in their jobs and needing to gossip with someone who would understand their difficulties. Within close relationships drivers told each other what they went through during the day, and gossiped about planners, clients, family. Close friends were perceived as best suited to offer emotional support, and venting about others with close friends helped drivers face daily stress and frustration experienced in the job.

*We ask what did you do, how was it? How did they speak to you, the people in the office? […]. We talk about what we have in our soul. I can’t talk about this kind of stuff by phone with my family, with my wife…. And there’s no one else more suitable. […] They understand because they are in the same situation as I am (R15).*

*We talk about what is bothering you. If you think they push you too hard, of course you say I wish they took it a bit easier. This planner is making me run around too much (R14).*

*Some clients act superior. They treat you like it’s your obligation to… like you are a slave. You get there and the fork lifter and the cleaning man is a boss. They don’t care about your schedule. […] We tell [each other], look what happened to me there. They kept me this long (R30).*

*If I don’t call him, he calls me, even 5 times [a day]. […] We talk about how we are, what we did back home, we talk about families too […]. We communicate, we advise each other what to do (R30).*

#### 4.2.2 Maintaining social bonds

Drivers reported that they often gossiped to keep track of friends, and to support each other if needed. Maintaining contact with people in their close network helped drivers gain a sense of community and belonging at work, which helped mitigate the effect of social isolation.

*Maybe one talked to another friend of mine before and then I don’t call him myself as well to see how he’s doing. The guy I’m talking with tells me about it (R5).*

*Those people who are from my region, I have many friends, so a lot of us work here from the region and it makes me feel better […]. We ask each other […] ‘Look, have you seen this guy?’ or ‘How was he doing? […] Only about those who are closer to me (R27).*

*For these people […] in my friend group […], four, five of us, we talk about each other. He is there, he is there, he is for the weekend there, somebody going home. If someone needs help (R28).*

*We are five or six guys [since] the first time we met with the truck and now I worry about that. Maybe he is in some trouble or something (R32).*

#### 4.2.3 Relaxation

Drivers also reported that gossiping with friends was an activity that helps them relax and detach from work. These conversations helped drivers cope by connecting with close others and bringing a sense of normalcy to their daily life. The accounts below illustrate this type of gossip.

*[We are] making jokes of other colleagues, yeah, every day! It’s normal […]. When you are making jokes it's… it releases you somehow, I don't know. Maybe releases my pressure 50% (R2).*

*We speak about private things, women, families. When I finish the job, I don’t speak about work (R6).*

Conversations between close friends had no clear time limit or topics that were strongly avoided (“high bandwidth,” Burt, 2001). Discussions between close friends organically integrated gossip.

*This is a good feeling for me […]. This is friendship [… and] very strong trust. We [are] talking about so many themes, so many issues, so many problems. Family problems, health problems, company problems, so many good news, and so many bad news (R25).*

To summarize, gossiping with others in one’s close circle of work friends allowed drivers to engage in emotion-focused coping. Such gossip helped manage stress, loneliness, and missing family and home, and had high intrinsic value for drivers by fostering connection and a sense of trust and belonging. By gossiping to their friends about others who have made their day difficult or about missing family, drivers offered each other emotional support. Furthermore, by helping drivers keep in touch with each other, gossip helped maintain strong bonds and a sense of community, in which they felt accepted and supported.

### 4.3 Gossip in distant relationships: problem focused coping

Drivers reported that they often talked with other drivers of the company whom they did not know well, or had not met before, and engaged in gossip with them. They encountered these colleagues at different locations. Among distant colleagues, gossip focused on exchanging useful information, and ended naturally after relevant information had been exchanged (i.e., extrinsic value). Gossip with distant colleagues provided drivers with essential information for understanding their work environment and its norms, enabling them to conduct their job better and improve safety.

#### 4.3.1 Establishing rapport

Drivers gossiped with distant colleagues in order to establish rapport, get to know others in the company, and understand the social network of the company. These conversations helped drivers gain a sense of their professional community and get acquainted with colleagues and planners working in the company. This type of information was not available by other means because drivers worked in isolation and getting to know each other was not facilitated by management.

*We always talk about the job. What you are doing, who is your planner, and for example, what job did you just have? (R27).*

*We ask [for gossip] but … not with a specific purpose. Only [to know] with whom he works (R29).*

*That’s why they get with each other sooner and quicker. And with this kind of questions like how much is your payment, and what is your boss and something like this (R2).*

*[…] next information is the client and the planners, who is the best planner, who is the worst planer. This is the most important information (R25).*

*[When] I first meet him who is he, when he started to work here, how it feels, what his impression is, and we exchange experience […]. Who is your planner, how it goes with him? Because we are the same, employees in this company, [and it is a] common subject for talking […]. How's that planner, we speak about that. Maybe in some time that planer will be my planner (R32).*

#### 4.3.2 Sense-making

By exchanging gossip about other people in the company drivers were able to gain information that helped them understand their own and others’ roles better, and what the company could legally ask of them. Gossip helped drivers understand the social norms in their company and how these may apply in their current situation. This type of gossip helped drivers gather information or insights not provided by the management.

*I heard that an older colleague went to Paris […] and he drove more than 10h. A motorcyclist went under him. The police came, […], although he wasn’t guilty of anything, it was the motorcyclist’s fault for speeding, but if he had died, the truck driver would have gone to prison, because he was the one who drove more than 10h. […]. And that’s why I said I’ll do like that. If something happens, the planner doesn’t come to answer in my place (R13).*

*I like to know about other situations, not just happy ones […], if something happened, if they heard [i.e., gossip about colleagues…]. Humans always learn from mistakes (R24).*

*[Gossiping] helps me at least to see that there is no way out. You must do what they say. Because otherwise… it's not that they would fire us […]. But we have to do it. If others do it, we have to do it as well (R18).*

Drivers often engaged in gossip with distant colleagues in order to hear news about other colleagues or managers and have a better understanding of their social environment.

*I exchange a few words, if they have any important information, I stay and listen, whether it’s good or bad. [… for example] that Ms. X left the company. Why? I am also curious about this (R29).*

*If someone gets sick, or has had an accident, or things like this, you hear about it in time. I hear from different colleagues. […] Truck driver stories always travel around. […]. It’s information, you know. Good, bad, at least you have information about what happened and how it happened (R5).*

Furthermore, drivers engaged in gossip with distant others to learn from others’ knowledge and experiences and compare these to their own, in order to verify information or check if their impressions were accurate.

*“In 2-3 days, this you can figure out [if someone told the truth]. We have our relations… I don’t even have to ask, and I find out” (I1).*

*They asked [for gossip] because maybe they [i.e., planners] annoyed him with something or asked him for something that made him uncomfortable. And then they ask me... Well, did this happen to me as well? (R26).*

#### 4.3.3 Solidarity

Drivers reported that they often gossiped out of a sense of solidarity with colleagues, to share with them useful information, in particular in about how to work with their planners.

*[I talk with colleagues] so they know what to expect. When they get to work with this planner, they will see what happens, or how it is. Maybe the person likes him, or maybe they don’t (R5).*

*Yes, I am always curious when someone is talking. I listen and I tell them my opinion as well. I even defended a colleague one day, who had trouble with the truck, and with the driving time. […]. And this bothered me, because even with no checks we don't necessarily have to go. We also get tired. It’s not important that there are no checks. We get tired either way. (R18).*

*I asked him […] ‘Did you send a message to the person in the office that you are waiting for one hour and cannot make it’? ‘No, but can’t he see it?’ ‘He probably doesn’t see, because he has 30 people [to keep track of] (R23).*

The company did not support drivers by providing information about how to solve typical problems they may face and did not provide training for new drivers. As illustrated below, drivers often helped each other learn important lessons by sharing their insights through gossip stories.

*I had a colleague who was in Switzerland and went in reverse in a tunnel. I said why? Why did you do such a stupid thing? […]. And I told the others “Don’t do what he did, go forward”. Not that I wanted to laugh at him, to drag him down, but to help others learn from it, and not do the same as him. Don’t put themselves in his situation… not regarding the fine and confiscating the truck. That doesn’t matter. But I am talking about his life, and the lives of others (R14).*

In summary, gossip with distant colleagues allowed drivers to engage in task-focused coping. By helping them access information and knowledge about colleagues and their planners, gossip improved their functioning at work. Due to working in isolation and with low support from management, drivers had restricted access to information and often experienced difficulties in completing their tasks or understanding how to solve problems. Therefore, gossip had an important informal mentoring role, allowing drivers to train and learn from each other, helping them solve problems and avoiding accidents and dangerous situations. Furthermore, by allowing drivers to become familiar with others in the company and to compare their observations and opinions, gossip had an important role in forming and validating perceptions of their professional community.

### 4.4 Communication (and gossip) avoidance in distant relationships

Besides the two patterns of gossip reported above, which were functional in coping with emotional and instrumental challenges arising from social isolation, results showed that in distant relationships drivers sometimes avoided gossiping with colleagues if these interactions did not provide useful information.

#### 4.4.1 Avoiding negativity and rumination

Drivers avoided gossip when their coworkers were perceived as unpleasant or toxic.

*They lie a lot […] about everything. […] Why should I speak with my colleague about the other colleague? […] Because they speak mostly ugly things. […] I really don't want to speak... you see me, in the corner [i.e. alone]. You can see when you go out you can see [other] drivers smoking and speaking (R7).*

Drivers also avoided gossip to detach from work focus on more pleasant activities.

*If you see another colleague and you only speak for [i.e., about] salary, for the tires for the fuel, for your boss […]. I don't like this information. I want to focus on myself. […] it's not my job to give advice [about other people] (R2).*

*When we sit here, I say guys, ok, I am preparing food, I don’t want to hear nothing about job. I have my free time. We can drink coffee beer, play volleyball (R11).*

Drivers were aware that gossip is often inaccurate and unreliable, and avoided drawing conclusions from it.

*There needs to be someone from the first source to describe the problem. That he was at X company, and they treated him badly, and this stuff. I cannot conclude anything from what he says about what happened there (R23).*

#### 4.4.2 Lack of shared background and identities

In distant relationships talking with each other was not interesting, because there was no meaningful connection between colleagues.

*In the beginning I had acquaintances, and even friends I grew up with were here in the company. But everybody left and now I'm the last one here. I have nothing to do with any of my colleagues (R9).*

*My opinion doesn’t matter, my advice. It is everyone’s business what they do back home, it’s their private matter. And I listen, but it gets boring. My thoughts run somewhere else (R29).*

Drivers reported that they had no common discussion topics with colleagues from different countries, because they had different mentalities and interests.

*When you are in a group, and no one talks to you, you can say anything and they don’t listen, you feel uncomfortable. You better leave (R1).*

W*hat shall I ask him? […] we have nothing in common except that we work in the same company. […] Among colleagues, I have no one. Given that they messed with the salaries, they [friends from own country] have left. [Now] there are colleagues from other countries. […] Now…it’s better alone (R14).*

*The indifference between people. Some time ago it wasn’t like that in this company, it was much better. We had better camaraderie. Now that is gone. […] Every nationality has a different mentality. A different understanding about everything (R5).*

Lastly, communication with foreign colleagues was difficult when employees did not speak any common languages.

*There are people who don’t speak any foreign languages, English or German, there’s no way to talk to them. Potentially use body language. There’s nothing you can do (R5).*

In summary, gossip avoidance emerged as a distinct pattern in distant relationships, that limited gossip exchanges with others with whom drivers did not feel connected.

## 5 Discussion

### 5.1 Gossip as a coping resource

The results revealed that gossip with close colleagues served primarily as a means of seeking emotional support and maintaining social bonds, which helped drivers cope with extreme loneliness and stress. Gossip was focused on sharing personal experiences drivers had during the day, their interactions with other people, and discussing concerns in depth (e.g., about relationships with managers, wellbeing of other close colleagues, family matters). Furthermore, drivers kept contact with others in their close network through gossip, which provided a sense of community and belonging, and helped them detach from work and relax. This form of gossip is consistent with previous theorizing and research which highlights the role of gossip in helping employees maintain social bonds, and gain a sense of shared reality, trust, and emotional support from others (Bosson et al., 2006; Beersma and Van Kleef, 2012; Peters et al., 2017). As such, talking with close colleagues was an essential coping mechanism because maintaining bonds with close others helped drivers reduce loneliness and overcome the effect of daily stressors (e.g., disrespectful clients, unsupportive managers). This improved their emotional well-being, possibly helping drivers stay in their jobs for longer (Demerouti et al., 2004; Schaufeli and Bakker, 2004).

Furthermore, findings showed that gossiping with distant colleagues represented a knowledge-sharing resource. In absence of other forms of contact due to the socially isolating nature of the job, gossip was essential for drivers in getting to know their professional community, helping them form and validate perceptions of coworkers and managers. Lacking other information sources, gossip ultimately helped drivers learn about and make sense of their social environment at work (Festinger, 1954). Furthermore, consistent with previous research, gossip helped drivers exchange essential information from which they could learn how to cope with current challenges (Fine and Rosnow, 1978; Baumeister et al., 2004; Beersma and Van Kleef, 2012; Martinescu et al., 2019). By sharing stories about others, drivers could access vital information for conducting their work safely and efficiently, which was not provided by the management. For example, drivers could learn about the (mis)adventures of other drivers, which contained rich lessons about how to solve typical problems or avoid failures, and what they could expect from managers. Thus, colleagues in the larger network mentored each other on how to best achieve work goals and deal with challenges of their job (e.g., safety). As such, gossiping in distant relationships provided access to new and diverse information (Burt, 2004). Gossip conversations with distant colleagues were perceived as having extrinsic value (i.e., a means to an end) and were instrumental in learning how to achieve work goals and gain practical knowledge, but ended after the useful information was exchanged. Thus, gossip with distant colleagues was instrumental in coping with the informational and managerial void encountered by drivers in their jobs by allowing drivers to engage in problem-focused coping.

### 5.2 Theoretical implications

First, despite working in isolation, drivers were part of a social network with multiple layers (Atkisson et al., 2020), as defined by the degree of closeness between people. Our findings indicate that the pattern of gossip between colleagues was substantially different in close and distant relationships, which had a primary role either in emotion focused coping or in problem-focused coping. As such, drivers could access different forms of social support from their strong and weak ties in the social network to cope with consequences of social isolation (i.e., loneliness, stress, knowledge limitations). This discovery is novel and a valuable contribution to JD-R model (Demerouti et al., 2004; Schaufeli and Bakker, 2004; Bakker and Demerouti, 2017), by clarifying how employees can draw support from their network via gossip in order to cope with job demands. These findings suggest that gossip should be seen as a resource, empowering employees to find the emotional or instrumental support they need to cope with job demands.

Second, in line with previous gossip research (Emler, 1994; Dunbar, 2004; Foster, 2004) our findings showed that gossip was pervasive in conversations between drivers. Although drivers worked in isolation, they gossiped abundantly with close and distant colleagues to meet their emotional and information needs. These findings extend previous research investigating the social functions of gossip (e.g., Baumeister et al., 2004; Bosson et al., 2006; Beersma and Van Kleef, 2012; Peters et al., 2017; Martinescu et al., 2019). Previous gossip research has mostly focused on contexts entailing higher goal interdependence between group members. Within such contexts, people form cooperative relationships on the basis of positive reputation, and they may gossip to warn others about those who behave badly or perform below group standards. In consequence, groups can eventually exclude people with a negative reputation, unless they conform to norms (Sommerfeld et al., 2007, 2008; Beersma and Van Kleef, 2011, 2012; Feinberg et al., 2014; Wu et al., 2016). However, in the current context, drivers gossiped abundantly about colleagues, clients, and managers, although this was not geared toward achieving shared work goals. Therefore, our unique research context helps bring to light adaptive functions of gossip that might remain hidden in typical work or social settings, where reputation processes play an essential role in shaping interactions. In the current research context, gossip had an adaptive role in maintaining supportive emotional bonds between community members and facilitating mentoring between peers; in absence of other organizational resources, gossip offered crucial resources that helped drivers cope with challenges deriving from social isolation at work.

Third, results also showed that gossip was not a universal coping solution. Sometimes gossip was avoided, especially in distant relationships, where no strong connections were present. As such, drivers experienced difficulties to establish meaningful connections with distant others, and they avoided gossip that did not provide useful information. Drivers had few opportunities to get to know their distant colleagues and develop closer relationships, and therefore perceived that non-instrumental communication had little value or was emotionally draining. This finding suggests that gossip is a somewhat costly coping strategy (Hobfoll, 1989; Hobfoll et al., 2018), because it requires time, energy, or patience (e.g., for overcoming language barriers or learning about dissimilar others). It is plausible that drivers might have been too depleted to sustain conversations with distant colleagues, and after exchanging useful information they preferred focusing on their existing social bonds or being alone. Drivers in our sample often reported that when their close friends left the company, they had difficulties forming new close relationships. Thus, distant social ties between drivers did not easily develop into closer ones over time. These insights show that in a context of limited contact, individuals have instrumental interactions to seek information or out of a sense of solidarity with their professional group, but they forfeit the opportunity to discover shared identities and develop closer bonds.

Fourth, our findings can be interpreted from the perspective of communicative constitution of organizations (CCO, Schoeneborn et al., 2019), which posits that communication (such as gossip) is the process that constitutes organizations. The findings of our current research are particularly aligned with this view – for the socially isolated drivers, the organization they worked for was construed through the gossip they participated in, which made possible knowledge exchange and filled information gaps. As a CCO process, gossip was essential in creating meaning and in defining the existence and nature of the organization (Waddington, 2022).

### 5.3 Limitations and future research

Our research has a number of limitations worth discussing. First, the work context of truck drivers is rather particular, therefore current findings should be interpreted within this context, and extended and validated in other settings. Our research was conducted in a context where employees experienced a specific set of challenges, in particular social isolation and work pressure. This study uncovered coping strategies used by people whose work is designed and managed based on principles of scientific management (Parker and Wall, 1998). Based on the current study, we cannot draw firm conclusions about whether the coping strategies we identified also occur in jobs where employees face different challenges. Future research in other contexts can help to determine to what extent current findings are generalizable. As such, future research should build on our insights by investigating the role of gossip as a coping mechanism in other work environments, where employees may face different challenges and relationships may have different dynamics.

Second, in more typical work settings, such as the business firm the gossip patterns we identified may also be observed, but perhaps these become more overlapping over time because employees have ongoing contact and have interdependent work tasks or goals, and therefore more dynamic social relationships. These two gossip patterns may be distinguishable especially in early stages of contact between employees and may change as colleagues get to know each other better. Some relationships may gradually shift from instrumental work relationships toward closer social relationships, and the gossip patterns with close and distant colleagues we discovered among truck drivers may become less clearly distinguishable. Future research could build on or findings to investigate how using gossip to cope with challenges may co-evolve with social relationships between people. Furthermore, research could investigate if the two gossip patterns identified here, emotion-focused and problem-focused, are associated with different gossip characteristics, such as gossip valence, level of formality, gossip confidentiality, and gossiper motives, which could further clarify how gossip is used in coping with different challenges.

Lastly, we are aware of potential methodological limitations of our study, such as social desirability bias and using an inductive, therefore subjective approach. Although these concerns are considered, we believe that the advantages of our methodological choices exceed their associated drawbacks. As a first study documenting the role of gossip as a coping mechanism with social isolation at work, we deemed an inductive approach most appropriate for capturing the ethos of the phenomenon, giving voice to our participants, whose perspectives we summarized and synthetized. Furthermore, we have no doubts regarding the sincerity and genuineness of responses, which were rich in raw emotions.

### 5.4 Practical implications

The extent to which employees need to rely on their social network of close and distant colleagues to cope with isolating work conditions could represent a barometer indicating how well organizations care for employees’ mental health and well-being, and enable them to perform their assigned tasks. From a management perspective, it is alarming when workers rely extensively on gossip with their colleagues to maintain their emotional well-being and gain role-related knowledge.

Based on our findings, we formulate practical recommendations that could improve the effects of working in a socially isolating environment. First, companies should become aware of the significant impact of social isolation on mental health (Apostolopoulos et al., 2016; Williams et al., 2017) and counteract this job demand by offering employees the opportunity to meet and interact with each other during social events. For truck drivers this would be feasible during their weekend breaks at the company base, which should occur more frequently. Second, in order to ensure that drivers have a stable social network of colleagues they can rely on and build strong bonds with, companies should adopt policies to prevent excessive turnover, and offer incentives for continued tenure. Third, managers should facilitate on the job training, and implement formal and informal programs through which employees can access vital information and knowledge, based on which they could work more effectively and safely. Exclusively relying on knowledge transfer via gossip networks is not optimal, because employees may miss opportunities to learn useful lessons. Training programs could be derived from the company’s vision on learning and development, allowing employees to grow into roles they feel drawn to, such as mentoring or coaching others, and getting recognition for their efforts.

Organizations may vary in the extent to which they are aware of how working conditions affect their employees. Arguably, some organizations may simply lack managers who are qualified to deal with non-technical challenges, and who are competent in designing work in a more human-centric manner. Others may purposely choose to ignore signals from employees or block communication channels that allow employees to voice their concerns. We emphasize the importance of organizations devising strategies to collect input from employees whose work socially isolates them from colleagues and managers, and implement systems of bottom-up feedback, allowing employees to voice their concerns and ask for support (e.g., pulse surveys, regular one-on one check-ins with a manager).

There is increasing societal attention to the responsibility companies bear in treating their employees well and enabling them to learn and develop in their roles. As such, we advise organizations focused on achieving profitability by maximizing the daily input of their labor force to consider whether the job design is sustainable and conducive to employee well-being and performance, and whether they could achieve profitability targets by supporting and training employees better.

### 5.5 Conclusion

Our research shows that a mix of two gossip patterns emerged as crucial in helping drivers cope well with the challenges of social isolation. Gossiping with close friends at work helped drivers engage in emotion-focused coping by reducing stress and loneliness, whereas gossiping with distant colleagues helped drivers engage in problem-focused coping by exchanging knowledge involving people in the organization. This study shows that gossip represents an essential resource for coping with job demands.

## Data availability statement

The raw data supporting the conclusions of this article will be made available by the authors, without undue reservation.

## Ethics statement

The study involving humans was approved by FSW Research Ethics Review Committee (RERC) of Vrije Universiteit Amsterdam. The study was conducted in accordance with the local legislation and institutional requirements. The participants provided their written informed consent to participate in this study. Written informed consent was obtained from the individual(s) for the publication of any potentially identifiable images or data included in this article.

## Author contributions

EM: Conceptualization, Data curation, Formal analysis, Investigation, Methodology, Project administration, Software, Supervision, Writing – original draft, Writing – review & editing. BB: Funding acquisition, Investigation, Project administration, Supervision, Validation, Writing – review & editing.

## Footnotes

^1^Participant nationalities and spoken languages are concealed in order to protect participants’ anonymity. Details can be obtained from the first author upon request.
